# Lactic acid is a sperm motility inactivation factor in the sperm storage tubules

**DOI:** 10.1038/srep17643

**Published:** 2015-12-01

**Authors:** Mei Matsuzaki, Shusei Mizushima, Gen Hiyama, Noritaka Hirohashi, Kogiku Shiba, Kazuo Inaba, Tomohiro Suzuki, Hideo Dohra, Toshiyuki Ohnishi, Yoshikatsu Sato, Tetsuya Kohsaka, Yoshinobu Ichikawa, Yusuke Atsumi, Takashi Yoshimura, Tomohiro Sasanami

**Affiliations:** 1 Department of Applied Biological Chemistry, Faculty of Agriculture, Shizuoka University, 836 Ohya, Shizuoka, Shizuoka 422-8529, Japan; 2 United Graduate School of Agricultural Science, Gifu University, 1-1 Yanagido, Gifu 501-1193, Japan; 3 Oki Marine Biological Station, Education and Research Center for Biological Resources, Faculty of Life and Environmental Science, Shimane University, 194 Kamo, Okinoshima-cho, Oki, Shimane 685-0024, Japan; 4 Shimoda Marine Research Center, University of Tsukuba, 5-10-1 Shimoda, Shizuoka 415-0025, Japan; 5 Research Institute of Green Science and Technology, Shizuoka University, 836 Ohya, Shizuoka, Shizuoka 422-8529, Japan; 6 Institute of Transformative Bio-Molecules (WPI-ITbM), Nagoya University, Furo-cho, Chikusa-ku, Nagoya 464-8601, Japan; 7 Graduate School of Bioagricultural Sciences, Nagoya University, Furo-cho, Chikusa-ku, Nagoya 464-8601, Japan

## Abstract

Although successful fertilization depends on timely encounters between sperm and egg, the decoupling of mating and fertilization often confers reproductive advantages to internally fertilizing animals. In several vertebrate groups, postcopulatory sperm viability is prolonged by storage in specialized organs within the female reproductive tract. In birds, ejaculated sperm can be stored in a quiescent state within oviductal sperm storage tubules (SSTs), thereby retaining fertilizability for up to 15 weeks at body temperature (41 °C); however, the mechanism by which motile sperm become quiescent within SSTs is unknown. Here, we show that low oxygen and high lactic acid concentrations are established in quail SSTs. Flagellar quiescence was induced by lactic acid in the concentration range found in SSTs through flagellar dynein ATPase inactivation following cytoplasmic acidification (<pH 6.0). The long-term preservation of sperm morphology under hypoxic and high temperature conditions indicates that a combination of these factors enables sperm cells to survive during the ovulation cycles. Our findings suggested a novel physiological role for lactic acid in promoting sperm quiescence in SSTs and opened up a new opportunity for technological improvement in prolonging sperm longevity at ambient or body temperature.

A diverse range of reproductive strategies have evolved in animals that all aim to achieve spatio-temporal regulation of the sperm-egg encounter during fertilization. In external fertilizers, such as sea urchins and ascidians, concurrent spawning of gametes from both sexes is common, and spermatozoa are known to employ chemotactic swimming towards spawned eggs by sensing the specific chemical gradients that they generate. The synchronized release of gametes in conjunction with sperm chemotaxis leads to more successful fertilization in external fertilizers^1^,^2^. In internal fertilizers, including mammals, successful fertilization also depends on the timely arrival of both sperm and egg at the site of fertilization. To achieve this, males transfer their spermatozoa to the female reproductive tract through copulation prior to or during ovulation. In a few species, such as rabbits and domestic cats, copulation itself stimulates ovulation^3^,^4^; however, in the majority of mammalian species, the timing of ovulation is not influenced by insemination. Females of these species are capable of storing spermatozoa in their reproductive tracts until their eggs are ready to be fertilized. This phenomenon is also common in many non-mammalian animals, including insects, fish, amphibians, reptiles, and birds^5^,^6^,^7^. Therefore, it can be concluded that sperm storage within the female reproductive tract is a common strategy utilized across several classes to ensure that sperm-egg interactions occur at the appropriate place and time^8^,^9^.

In avian species, sperm storage tubules (SSTs), simple tubular invaginations located between the vagina and uterus, serve as sperm storage sites^10^,^11^. Once ejaculated, spermatozoa migrate to and stay in the lumen of SSTs, where they can remain with fertilizability for long periods of time (up to 15 weeks) at a normal body temperature of 41 °C^12^. Researchers have questioned how sperm can survive and remain capable of fertilizing under these storage conditions without the ability to synthesize proteins. Sperm storage in SSTs is an essential step for fertilization in birds, as SSTs protect sperm from noxious conditions, such as anti-sperm immune responses^13^, vaginal sperm ejection^14^, and mechanical flushes caused by egg extrusion; therefore, it is believed that only spermatozoa successfully stored in SSTs are capable of fertilizing ova in domestic birds.

Of particular interest is that avian spermatozoa are able to undergo the transformation from quiescent to active within the female reproductive tract twice, i.e., after the male’s ejaculation and also after release from the SSTs^15^. Previous studies have explored the physiological roles of SSTs in sperm quiescence^16^,^17^,^18^; however, the underlying molecular mechanism of this process has remained a mystery. In this study, we hypothesized that SSTs contain a substance(s) that renders sperm motility inactive. Based on this hypothesis, we aimed to identify the component(s) responsible for sperm quiescence within SSTs.

## Results

### Identification of the sperm inactivation factor in the SSTs

**Figure 1:**
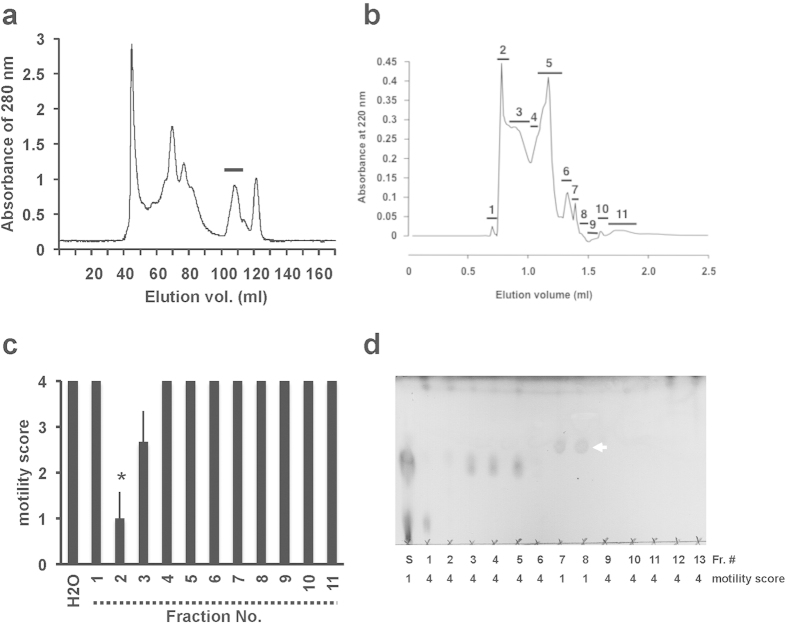
Purification of a bioactive substance from utero-vaginal junction extracts. (**a**) Utero-vaginal junction (UVJ) extracts were loaded onto a Superdex 200 pg column. The detectable peak was collected and each fraction was evaporated to dryness, dissolved in a physiological saline (pH 7.4), and applied to the sperm motility assay. The bioactive fraction is indicated by the horizontal bar. (**b**) HPLC profile of bioactive fractions (in panel a) on a C-22 reverse-phase column. Each collected fraction is indicated with a horizontal bar and a numeral. (**c**) Effects of HPLC fractions on sperm motility. Ejaculated sperm were suspended in a medium containing each fraction (1% (v/v)) and the effects of each fraction on sperm motility were evaluated. Bioactivity was detected in fractions 2 and 3. Asterisk indicates a significant difference from H_2_O. (**d**) Thin layer chromatography (TLC) analysis of the bioactive substance. A mixture of fractions 2 and 3 (in panel b) was further separated through preparative TLC. The motility score for sperm in each fraction are indicated below the TLC photograph. White arrow indicates the bioactive substance. S, mixture of fractions 2 and 3.

**Figure 2:**
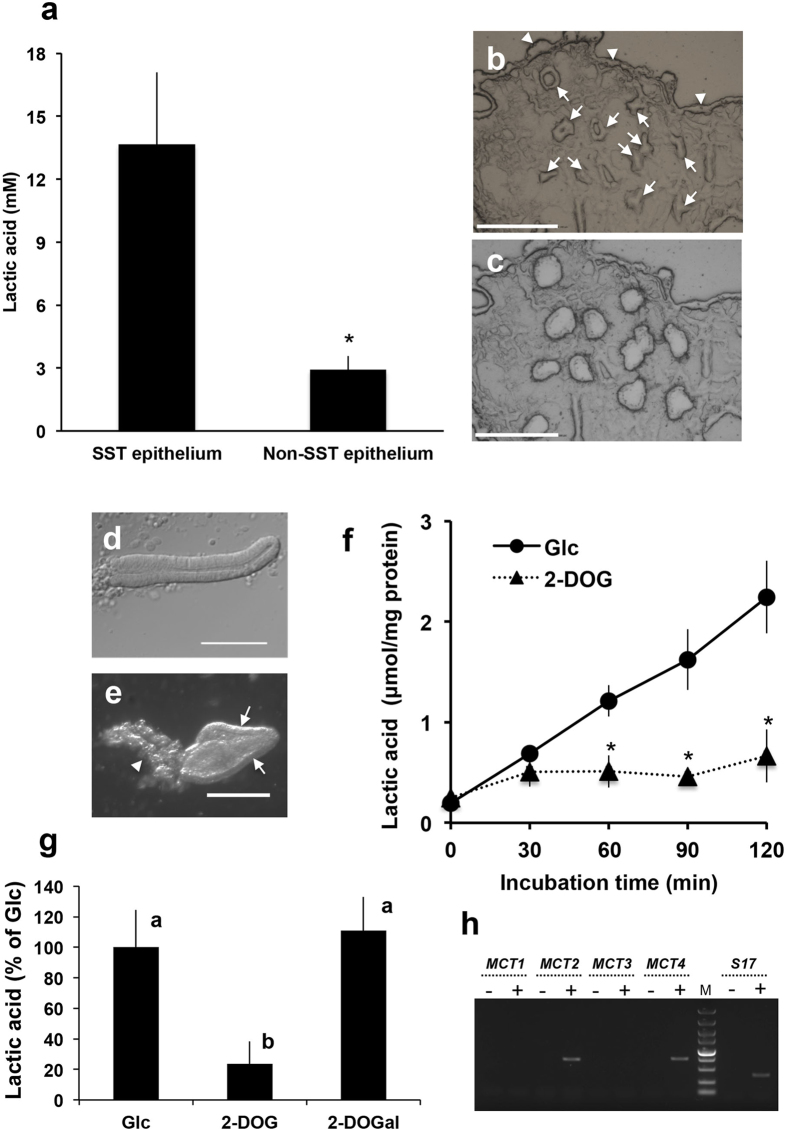
Sperm storage tubules produced large quantities of lactic acid. (**a**) Lactic acid levels in sperm storage tubule (SST) epithelium extracts and surface epithelial cells (Non-SST epithelium) isolated by laser microdissection (LMD). Values shown are means ± SEM of four independent experiments. Lactic acid levels were significantly greater in the SST epithelium than in the Non-SST epithelium (P < 0.05). (**b**,**c**) Photographs of a cryosection of the utero-vaginal junction (UVJ) before (**b**) and after (**c**) LMD. Arrows and arrowheads in panel b indicate SSTs and Non-SST epithelium, respectively. Scale bar = 150 μm. (**d**) Photograph of SSTs isolated by collagenase digestion. Scale bar = 100 μm. (**e**) Photograph of non-SST cells isolated by collagenase digestion. Scale bar = 200 μm. Arrows and the arrowhead indicate surface epithelial cells and lamina propria, respectively. (**f**) Time-dependent release of lactic acid by cultured SSTs. Isolated SSTs were cultured in Hank’s balanced salt solution containing either 5 mM glucose (Glc) or 1 mM 2-deoxyglucose (2-DOG). The medium was sampled at the indicated times and the lactic acid levels of the samples were measured. Values shown are means ± SEM of three independent experiments. Asterisks indicate significant differences from Glc at each incubation time (P < 0.05). (**g**) SSTs produced lactic acid via glycolysis. Isolated SSTs were cultured in Hank’s balanced salt solution containing either 5 mM glucose (Glc), 1 mM 2-deoxyglucose (2-DOG) or 1 mM 2-deoxygalactose (2-DOGal). The medium was sampled at 60 min of incubation and the lactic acid levels of the samples were measured. Values shown are means ± SEM of three independent experiments. Different letters denote significant differences (P < 0.01). (**h**) Expression of monocarboxylate transporter mRNA. mRNA extracted from the UVJ was reverse transcribed, and an aliquot was subjected to PCR using the primer set (*MCT1*, *MCT2*, *MTC3*, *MTC4* or *S17*) indicated at the top of the figure. For a non-RT control, mRNA from the UVJ was treated the same way, except reverse transcriptases (lanes -) were omitted. The representative gel of three independent experiments was shown. M, molecular weight marker.

Tissue from the utero-vaginal junction (UVJ), an area where SSTs are present, was homogenized and ultrafiltrated (>10 kDa cutoff) to obtain UVJ extracts. The flow-through fraction was found to strongly suppress sperm motility *in vitro* (Supplementary Movies 1 and 2), whereas the fraction retained on the ultrafiltration membrane (i.e. >10 kDa) had no significant inhibitory effect on sperm motility; thus, the flow-through fraction was pooled for subsequent procedures. The bioactive substance was further purified by liquid chromatography using size-exclusion (Fig. 1a) and C-22 reverse phase (Fig. 1b,c) columns followed by preparative thin-layer chromatography (TLC) (Fig. 1d). Analytical TLC of the sample in the final step of purification detected only one major spot (Fig. 1d, *arrow*), which was thereafter assigned as lactic acid by the spectrum analysis (see *Methods*). To determine the concentrations of L-lactic acid in SSTs, we used laser microdissection to isolate SST epithelial cells and non-SST epithelial cells (the ciliated epithelial cells that cover the surface of the UVJ) from frozen sections of UVJ. This method minimizes loss of small molecules. Although the epithelial lining of the SSTs or the surface epithelium was not clearly visible on the frozen sections without fixation and staining, the SSTs were easily distinguished by a unique ring- or tube-like structure (Fig. 2b). Extracts of the epithelial cells were assayed for L-lactic acid, which was found to be five times greater in SST epithelial cells (14 ± 3.4 mM, n = 4) than in non-SST epithelial cells (2.9 ± 0.6 mM, n = 4, Fig. 2a–c).

### Mechanism for the release of a large amount of lactic acid from SSTs

To investigate the mechanism of lactic acid accumulation in the SSTs, they were isolated from the UVJ by collagenase digestion (Fig. 2d) and cultured *in vitro* in a glucose-containing medium. The non-SST cells isolated by collagenase digestion consisted of surface epithelial cells and lamina propria. The representative photograph of the non-SST cells is shown in Fig. 2e. The concentration of lactic acid in the SSTs culture medium increased markedly with time (Fig. 2f). This increase was completely lost by the addition of 2-deoxy-D-glucose, an inhibitor of glucose metabolosm (Fig. 2f)^19^, but not by the inactive analog, 2-deoxy-D-galactose (Fig. 2g). Furthermore, reverse transcription polymerase chain reaction (RT-PCR) with SST-isolated RNAs identified transcripts encoding the monocarboxylate transporters 2 and 4 (*MCT2*, *MCT4*), which are responsible for lactic acid uptake and lactic acid release, respectively^20^,^21^ (Fig. 2h). Presence of the transcripts for *MCT4* in the SSTs was also confirmed by gene-specific *in situ* hybridization (Supplementary Fig. S1 online). Together, these results indicated that lactic acid was produced in SST cells through the glycolytic pathway and may be released via MCT4. However, the tissue localization of MCT2/4 as well as the metabolic pathways that give rise to L-lactic acid should be identified in future studies.

**Figure 3:**
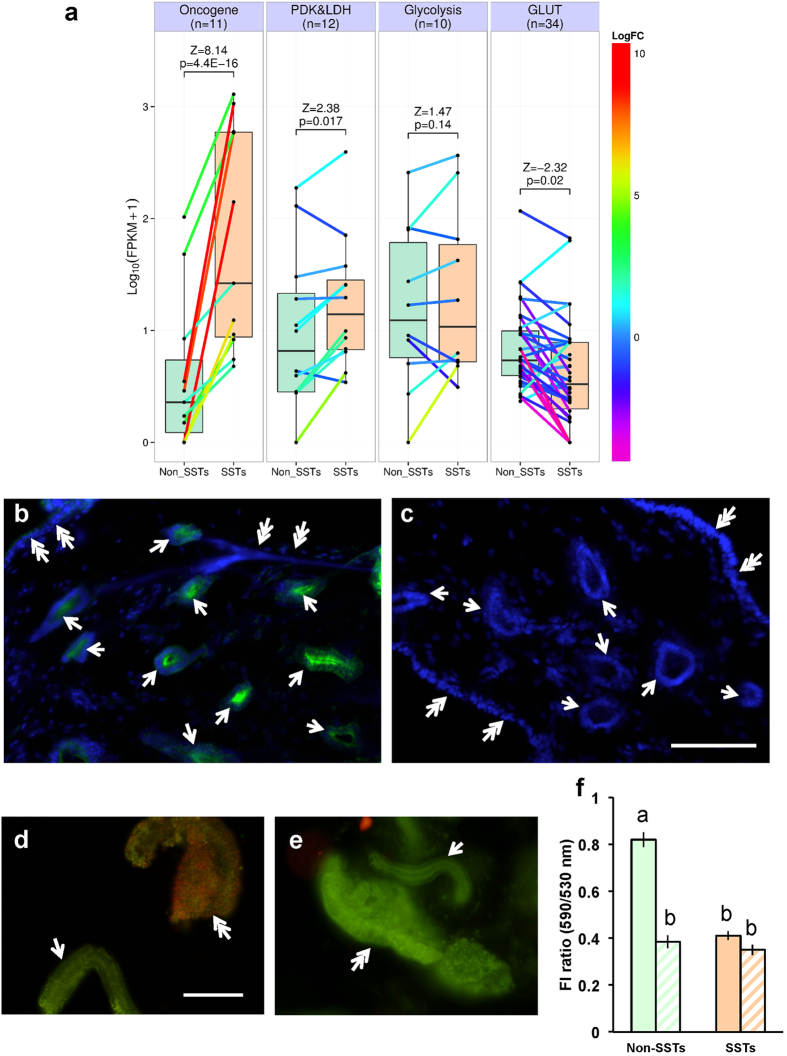
Sperm storage tubules were subjected to hypoxic conditions. (**a**) Differential gene expression between the non-SST cells and SST cells. Box plots represent log_10_(FPKM + 1) gene expression levels for the functional gene sets related to oncogene, pyruvate dehydrogenase kinase and lactate dehydrogenase (PDK&LDH), glycolysis, and glucose transporter (GLUT) in non-SST cells (Non_SSTs) and SST cells (SSTs). Colored lines between the gene expression values in Non_SSTs and SSTs represent log_2_-fold changes (LogFC), with values indicated on the color scale to the left. n, numbers of genes in the functional gene sets; Z and p, Z scores and p-values, respectively, calculated by parametric analysis of gene set enrichment based on the logFC between Non_SSTs and SSTs. (**b,c**) Immunohistochemical detection of hypoxic cells. UVJ tissues isolated from birds injected with Hypoxyprobe^TM^. (**b**) or saline (**c**) were stained with anti-pimonidazole antibody (green). The nuclei were counterstained with 4′,6-diamidino-2-phenylindole (DAPI; blue). Arrows and double arrows indicate SSTs and surface epithelium, respectively. Scale bar = 100 μm. (**d,e**) Mitochondrial activities of SSTs and surface epithelium assessed by JC-1 fluorescence in the presence (**e**) or absence (**d**) of an uncoupler. Surface epithelium displayed high mitochondrial activities (double arrow in panel (**d**)) while low mitochondrial activities were exhibited by SSTs (arrow in panel (**d**)). On the other hand, differences in mitochondrial activities between surface epithelium (double arrow in panel (**e**)) and SSTs (arrow in panels (**e**)) were absent in the presence of an uncoupler (panel (**e**)). Scale bar = 100 μm. (**f**) FI ratio (590 nm/530 nm) in the presence (hatched bars) or absence (solid bars) of an uncoupler. FI ratio indicated mitochondrial activity. Mitochondrial activities of SSTs were significantly lower than those of surface epithelium (Non-SSTs). Values shown are means ± SEM of three independent experiments. Different letters denote significant differences (P < 0.0004).

Next, the gene expression profile related to glycolysis was assessed using RNA-seq to infer the mechanism for the massive production of lactic acid. The data set obtained in this study has been deposited in the DDBJ Sequence Read Archive (http://trace.ddbj.nig.ac.jp/dra/index.html) under accession number DRA003919. Unexpectedly, the expression levels of key glycolytic enzymes, such as hexokinase, phosphofructokinase, and pyruvate kinase, as well as several glucose transporters were not significantly higher in SSTs than those in non-SST cells, suggesting that enhanced glycolysis is not the primary mechanism in the mass synthesis of lactic acid in the SSTs (Fig. 3a). In accord with this, a KEGG PATHWAY mapping analysis (http://www.genome.jp/kegg/pathway.html) with the SST transcriptome dataset revealed no sign of enhanced glycolysis. Analysis of differential gene expression profiles with several sets of gene ontology (GO) between SST cells and non-SST cells failed to uncover molecular evidence for the mass production of lactic acid in the SSTs (Supplementary Table S1 online). In contrast, we found a dramatic increase in the expression of *c-Fos* and *c-Jun* in SSTs compared to non-SST cells (Fig. 3a). As it is known that these oncogenes are upregulated in highly proliferating cells^22^ and in cancer cells under hypoxic conditions^23^, and hypoxia has been shown to be an alternative factor promoting lactic acid production in cells^24^, we hypothesized that in physiological condition, SST cells are subjected to hypoxia. To test this hypothesis, Hypoxyprobe^TM^ was used to detect hypoxic cells and tissues through immunohistochemistry. We found that the intensity of the hypoxic signal was much greater in SST cells than non-SST cells (Fig. 3b,c). In addition, a marked decrease in mitochondrial activity within SST cells supported the idea that oxygen is limited in SSTs (Fig. 3d,e,f). From these results, we concluded that hypoxia is the likely cause for the accumulation of lactic acid in SSTs.

### Mechanism of sperm inactivation by lactic acid

**Figure 4:**
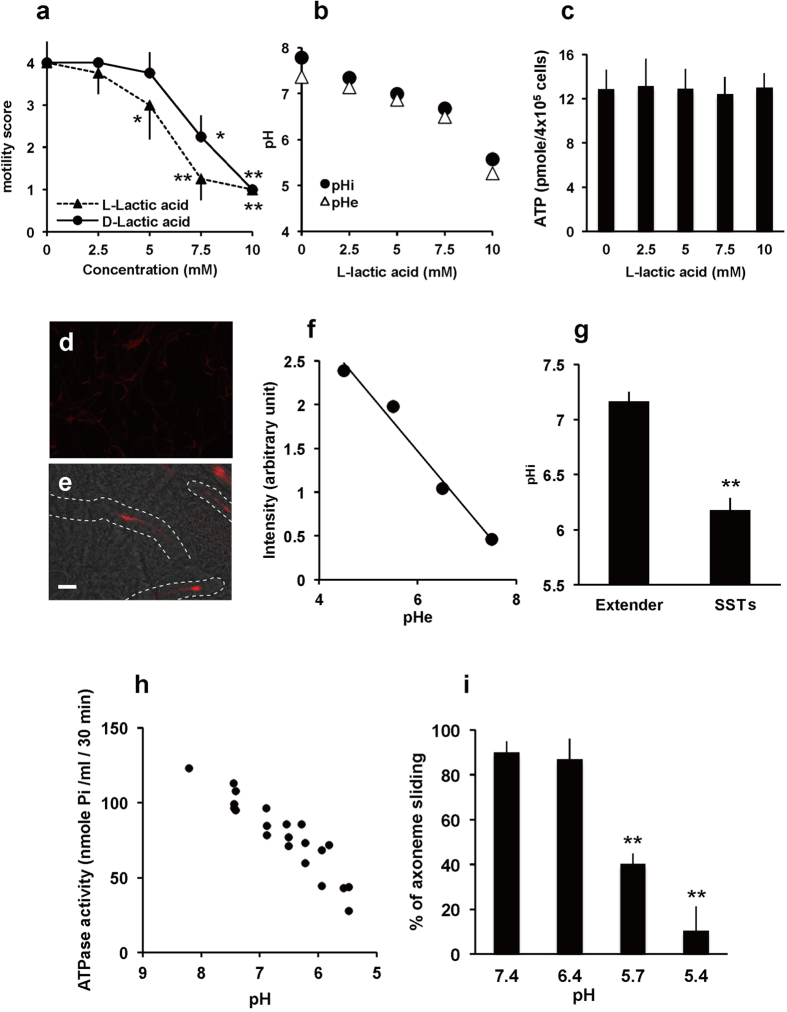
Mechanism of sperm motility inactivation that resulted from exposure to lactic acid. (**a**) Effects of lactic acid on sperm motility *in vitro*. Ejaculated sperm were suspended in media containing various concentrations of either L-lactic acid or D-lactic acid, and sperm motility was evaluated on a scale of 0 to 5. Values shown are means ± SEM of three independent experiments. *P < 0.05 vs. 0 mM. **P < 0.01 vs. 0 mM. (**b**) Changes in the pH_e_ or pH_i_ of sperm in response to the addition of L-lactic acid. pH_e_ of the medium was directly measured using a pH meter. Sperm pH_i_ was determined using pHrodo^TM^. The results shown are representative of results from five independent experiments. (**c**) ATP levels in sperm incubated with L-lactic acid. ATP concentrations of sperm are expressed as mean values ± SEM of three independent experiments. (**d**,**e**) Photographs of pHrodo^TM^-loaded sperm swimming in Hank’s balanced salt solution (**d**) and resident sperm in SSTs (**e**). Dashed line indicates the outline of SSTs. Scale bar = 50 μm. (**f**) Representative standard curve of sperm pH_i_ equilibrated with the indicated pH buffer containing valinomycin and nigericin. (**g**) pH_i_ values of sperm swimming in Hank’s balanced salt solution (Extender) and resident sperm in SSTs (SSTs). Values shown are means ± SEM of three independent experiments. Asterisk denotes a significant difference (P < 0.00001). (**h**) Effects of pH on ATPase activity of sperm. De-membraned sperm were incubated with 1 mM ATP, and the free phosphoric acid in the incubation mixture was measured using a microplate reader. (**i**) Effects of pH on axonemal sliding of sperm. Demembraned sperm were mounted on chamber slides, treated with trypsin, and an assay buffer that adjusted the pH to either 7.4, 6.4, 5.7, or 5.4 was perfused for equilibration. The ATP solution was perfused and axonemal sliding was recorded. Percentages of axonemal sliding were calculated and expressed as mean values ± SEM of three independent experiments. **denotes a significant difference from pH 7.4 (P < 0.0000001).

The effects of exogenous lactic acid on sperm motility were then investigated *in vitro*. Sperm motility decreased in response to exposure to L-lactic acid in a dose-dependent manner, while D-lactic acid had a lesser inhibitory effect (Fig. 4a and Supplementary Movie 3), suggesting the involvement to some extent of molecules with stereospecificity such as lactate/pyruvate dehydrogenases. However, the sperm quiescence activity was not due to lactic acid itself because the addition of other organic acids such as acetic acid, malic acid, oxaloacetic acid, citric acid and even hydrochloric acid (pH 5) inactivated sperm motility (Supplementary Fig. S2 online). Notably, a strong correlation between extracellular pH and sperm quiescence was observed (R^2^ = 0.95, Supplementary Fig. S2 online), suggesting that the sperm immobilization is caused by lowering extracellular pH (pH_e_). The data showed that 10 mM lactic acid was sufficient to immobilize sperm, which is in line with the physiological concentrations found in SST extracts (i.e., 14 ± 3.4 mM). Because pH_e_ decreased in response to the addition of lactic acid (Fig. 4b), we next examined whether lactic acid addition can decrease sperm intracellular pH (pH_i_). When pHrodo-loaded sperm were exposed to different concentrations of L-lactic acid, sperm pH_i_ and pH_e_ were concurrently decreased (Fig. 4b). On the other hand, intracellular ATP levels were not affected by the addition of L-lactic acid (Fig. 4c), which indicated that the lactic acid-induced sperm quiescence is not due to the depletion of intracellular ATP. Furthermore, a direct pH_i_ measurement of SST-stored sperm, which was enabled by intra-vaginal insemination of pHrodo-loaded sperm, clearly demonstrated that intracellular acidosis of the resident sperm occurred (Fig. 4d,e,g, pH 6.2 ± 0.1, means ± SE, n = 29).

These findings suggested that the lactic acid-induced cytoplasmic acidification plays a key role in sperm quiescence in SSTs. It is well known that dynein-mediated sliding of the axonemal outer doublet microtubules drives flagellar movement of sperm, and that the energy required for this motility is provided by the ATP hydrolysis that results from the activity of dynein ATPase^25^. In sea urchin sperm, ATPase activity has a narrow window of pH optima (between 7.5 and 8.6) and the activity decreased rapidly if pH gets out of this range^26^. Therefore, we investigated whether the ATPase activity of quail sperm was affected by pH. As shown in Fig. 4h, the ATPase activity of demembraned sperm rapidly decreased along with the decreasing pH. At pH 6.0, which was equivalent to the pH_i_ of resident sperm, the ATPase activity of sperm was <50% than that of sperm at pH 8.0. In the positive control, where sodium vanadate, a selective inhibitor for dynein ATPase was added, ATPase activity was significantly decreased. Conversely, this decrease was not observed in the presence of oligomycin, an inhibitor for F_o_ ATPase of mitochondria (Supplementary Fig. S3 online). The percentage of quail sperm that exhibited axonemal sliding also decreased in a pH-dependent manner (Fig. 4i and Supplementary Movies 4 and 5). These results strongly supported the hypothesis that sperm flagellar quiescence resulted from lowered sperm pH_i_ caused by the lactic acid accumulation in the SSTs. The assertion that hypoxia plays a role in the process of sperm storage was further supported by improved morphological preservation of ejaculated spermatozoa under hypoxic conditions at body temperature (Supplementary Fig. S4 online), which indicated that hypoxia may potentiate the preservation of sperm fertilizability within SSTs.

## Discussion

After ejaculation, sperm are transferred into the vagina, become motile, and a small population of sperm enters the SSTs within 1 hr of copulation^27^. Although the mechanism underlying the sperm transport into SSTs is not known, it is reported that vigorous sperm motility is required for sperm invasion into SSTs. Nevertheless, once sperm have entered into the SSTs, they become quiescent in terms of motility by an unknown mechanism(s) and are stored until use for fertilization. To our knowledge, the present study is the first to report the massive production and release of lactic acid from SSTs under a hypoxic condition leading to the inactivation of resident sperm. We provided evidence for cytoplasmic acidification caused by luminal lactic acid accumulation playing a key role in sperm quiescence in SSTs. This suggests a novel physiological function of lactic acid in vertebrates as a key regulator in sexual reproduction. Because sperm inactivation was also induced by extracellular acidification by other organic acids as well as HCl, lowering pH in the lumen of the SSTs is important for sperm inactivation. In the SST, the bulk release of lactic acid from the cells, which occurred under the hypoxic condition, is inevitably used for sperm quiescence. Our result showed that sperm intracellular acidosis occurred concomitantly with the decrease of pH_e_, indicating that extracellular protons might pass through the plasma membrane of spermatozoa via an unknown proton transporter/channel. Although direct evidence is lacking, MCTs do not likely contribute to intracellular acidosis because malic acid (dicarboxylate) and citric acid (tricarboxylate) also inactivated sperm motility. The transporter/ion channel responsible for the proton transport remains to be identified in future studies.

In the lumen of SSTs, sperm are typically immotile^28^,^29^, and thus regarded to be metabolically quiescent as a result of a lowered ATP consumption rate. This is also favorable for sperm survival because it leads to a reduced production of reactive oxygen species by sperm respiration. A similar mechanism for sperm quiescence is reported in the mammalian epididymis where luminal acidification, achieved by the bulk of proton secretion from the specialized epithelial cells through apically-localized vacuolar proton pumping ATPase, plays a role in sperm motility inactivation during storage^30^. Thus, a decrease in pH_e_ could play a universal role in the process of sperm quiescence. However, another model has been suggested in the chicken, in which an outward fluid stream was generated in the SSTs and the sperm maintained their position against this stream by their own forward power generated by flagellar movements^16^. In this model, sperm efflux from the SSTs is thought to be the result of reduced sperm velocity due to a shortage of energy supplementation from sperm mitochondria. In contrast, our previous study in the quail demonstrated that progesterone stimulates the release of the resident sperm from the SST because most of the sperm that resided in the SSTs were released by contraction-like morphological changes of the SSTs within 1 hr of progesterone injection^31^. Although the reasons for this discrepancy between chicken and quail are not known, Bobr *et al.* who investigated the distribution of spermatozoa in the hen oviduct reported that the resident spermatozoa were discharged from the SSTs close to the times of ovulations and/or ovipositions^32^. These results indicated that the timely release of sperm from SSTs could be controlled by hormone in the chicken.

In this study, we were unable to produce fertilized eggs by artificial insemination of the female birds using *in vitro* stored sperm, indicating that sperm preservation requires unknown factor(s) other than lactic acid. It is reported that the resident sperm agglutinate “head-to-head” in the SSTs of the chicken^33^,^34^. Our previous observation in the quail using light or electron microscopy demonstrated that most of the sperm in the SSTs make contact with each other in a uniform bundle-like agglutination, and single sperm were seldom seen^27^. Although the mechanism of sperm agglutination is unknown, we expected that a characteristic of sperm agglutination might be another basis for prolonged *in vivo* storage of spermatozoa because this mode of sperm residency is common among domestic birds^27^,^28^,^29^,^31^. In addition, ultrastructural observations in turkey SSTs revealed the presence of membrane fragments and small vesicles in the lumen of the SSTs, some of which appeared to have fused with the plasma membrane of the resident sperm^35^. Such interactions also likely to contribute to prolonged sperm storage in the SSTs. The current knowledge in the process of sperm storage is limited. For instance, the main unanswered questions are; “How do the ejaculated spermatozoa enter the SST?”; Which molecule contributes to sperm-sperm contact?” and “What stimulus triggers the release of this assembly?” Understanding of such the mechanisms will contribute to the development of the artificial sperm storage technology. Further studies are needed to uncover the mechanism of sperm storage in avian species.

Understanding the cellular and molecular mechanisms of the avian sperm receptacle system could provide insights into the development of novel methods for sperm storage at ambient or body temperatures, which would prove beneficial to assisted reproductive therapies, such as artificial insemination and *in vitro* fertilization.

## Methods

### Animals and tissue preparation

Eight to 20 week-old Japanese quail, *Coturnix japonica* (Motoki Corporation) were maintained individually under a photoperiod of 14 L: 10D (lights went on at 05:00) with *ad libitum* access to water and a commercial diet (Motoki Corporation). The utero-vaginal junction (UVJ) mucosa around the junction of the uterus and the vagina were dissected out and placed in physiological saline. The UVJ mucus membranes containing SSTs were isolated with forceps and scissors under a stereomicroscope according to the method of Ito *et al.*^27^.

Semen was obtained from male quail during mating prior to ejaculation according to the procedure of Kuroki and Mori^36^. Semen obtained from two to three males was suspended in a sperm extender (136 m mol/l NaCl, 5.4 m mol/l, KCl, 0.8 m mol/l MgSO_4_, 1.26 m mol/l CaCl_2_, 4.2 m mol/l NaHCO_3_, 5.6 m mol/l glucose buffered at pH 7.4 with 10 m mol/l HEPES). The sperm concentrations were measured with a hemocytometer and sperm were incubated at 39 °C in all experiments. All experimental procedures for the care and use of animals were carried out in accordance with approved guidelines of the Animal Care Committees of Shizuoka University (Approval number: 26–12).

### Extraction and purification of sperm inactivation factor

Excised quail UVJ tissues were minced in 20 mM ammonium formate (pH 7.4, 1 g wet tissues in 2 ml buffer) and extracted for 3 hr on ice with occasional shaking. The extracts were centrifuged at 20, 000 *g* for 10 min at 4 °C and the supernatant lyophilized to dryness. The dried sample was dissolved in 20 mM ammonium formate and filtered through a membrane (0.45 μm: Millipore). The filtrate was subjected to separation on a Superdex 200 pg column (GE Healthcare) according to our previous methods^37^. The bioactive fractions were pooled, lyophilized and dissolved in water. This material was loaded onto a C-22 reversed-phase column (Wako pure chemicals) and eluted with water. The bioactive fractions were then applied onto a preparative thin layer chromatography (TLC, Merck) with ethyl acetate: methanol: water = 6: 4: 1 as a solvent. Every 1 cm silica gel was shaved off from the TLC plate and extracted with the same solvent. The extracted materials were evaporated to dryness and dissolved in water. The purity of the bioactive substance was confirmed by TLC analysis with the same solvent.

### Structural analysis of bioactive substance

^1^H NMR, ^13^C NMR and 2D NMR (HMQC and HMBC) spectra were observed with a JEOL GSX-500 spectrometer at 500 MHz in D_2_O. The spectroscopic data of the isolated compound were compared with a reference compound (L-lactic acid, Sigma). Based on the structure analyses, the substance was identical to lactic acid.

### Isolation of SST

SSTs and non-SST samples were isolated with either collagenase treatment or microdissection. Collagenase treatment is suitable for RNA preparations due to the short time process, whereas microdissection is suitable for recovering small molecules such as lactic acid. SSTs were isolated from UVJ mucosa as in the method of King *et al*^38^. Briefly, the UVJ mucosa was placed in collagenase solution (10 mg/ml collagenase (Wako pure chemicals) in Hanks balanced salt solution (HBSS)) in the ratio of 1 mg wet weight/μl and minced into small pieces by scissors. Collagenase solution (typically 400–500 μl) was further added and incubated for 20 min at 37 °C with occasional shaking. Collagenase treatment was terminated by adding 5 ml of ice-cold Ca^2+^/Mg^2+^ -free HBSS containing 10 mg/ml bovine serum albumin. The resulting preparation was transferred to a Petri dish, and the dissociated SSTs were collected by a micropipette under a stereomicroscope (M165FC; Leica microsystems). The non-SST cells were also collected from the same preparation under a stereomicroscope. The isolated SSTs were then incubated in HBSS with or without 5 mM glucose, 1 mM 2-deoxy glucose (Wako pure chemicals) or 1 mM 2-deoxy galactose (Wako pure chemical). The culture supernatant was recovered by centrifugation at 20,000 × *g* for 10 min at 4 °C. The concentration of L-lactic acid in the medium was determined as below.

### Measurement of lactic acid

The birds were decapitated, the UVJ was removed, and 30 μm frozen sections were prepared. The SST or the surface epithelium was captured by laser microdissection (PALM Combi system, Zeiss), and the total volume of the tissues obtained was calculated. 100 μl of water was added and homogenized by a Polytron. The homogenate was centrifuged at 20,000 × *g* for 10 min at 4 °C and the supernatant evaporated to dryness. The dried extract was dissolved in water and the concentration of L-lactic acid was determined by an enzyme-based L-lactate assay kit (Cayman), and calibrated by the total volume of the tissues. The enzymatic reaction was not interfered with D-lactic acid, pyruvate, phosphoenolpyruvate, 2-oxobutyrate or oxaloacetate, up to a final concentration of 1 mM. The inter- and intra assay coefficients of variation were 2.95% and 3.65%, respectively.

### Sperm motility analysis and intracellular pH measurement

Ejaculated sperm were incubated with a sperm extender containing bioactive fractions or various concentration of L-lactic acid (Sigma). An appropriate vehicle was included in the incubation mixture for the control experiments. Motility was evaluated by observing the sperm in several areas of the petri dish directly using a stereomicroscope and their motility was scored on a scale of 0–5 as in Wheeler and Andrews^39^.

For intracellular pH (pH_i_) measurement, the ejaculated sperm (2 × 10^7^ cells/ml) were incubated with 1 μM pHrodo-AM, a fluorescent pH_i_ indicator (Molecular probe) for 10 min at 39 °C. After the incubation, the sperm suspension was added to the sperm extender that was supplemented with or without L-lactic acid or D-lactic acid and incubated for an additional 10 min. Sperm pH_i_ was determined by spectrofluorophotometer (RF-5300PC, Shimadzu). In the case of the artificial insemination (AI), the sperm incubated with pHrodo-AM were washed two times with PBS by repeated centrifugation at 800 × g for 3 min, and the final pellet was intra-vaginally injected. After 1 hr of the AI, the UVJ was isolated as described above. After washing with PBS, the specimens were fixed with a 3.7% formaldehyde solution, and the fixed UVJ was cut into small pieces with scissors, mounted in glycerol and photographed under a fluorescence microscope (BX 51, Olympus) with a 20x objective (UplanApo20X, NA0.70; BX 51, Olympus). The fluorescence intensity of the sperm head was digitized using an image analysis system (ImageJ v. 1.440, http://imagej.nih.gov/ij). The pH_i_ was standardized by intracellular pH calibration kit as suggested by the manufacturer (Molecular Probes). The pHe value was obtained by the direct measurement of each medium’s pH using a pH meter (LAQUAtwin, HORIBA Scientific).

### Evaluation of mitochondrial activity

The mitochondrial activity of the SSTs and the surface epithelium was evaluated by staining with JC-1 (5,5′,6,6′-tetrachloro-1,1′,3,3′-tetraethylbenzimidazolylcarbocyanine iodide, Molecular Probes). When the membrane potential of the inner mitochondrial membrane is high, indicating an active state, JC-1 emits orange fluorescence (590 nm), while green light (530 nm) is produced at low membrane potential. The UVJ mucosa was minced in 50 μl HBSS and a 500 μl JC-1 solution diluted in HBSS (final concentration, 125 nM) was added into the test tube. After loading for 20 min at 37 °C, the UVJ was collected by centrifugation at 800 × g for  min and washed with 1 ml HBSS for 20 min at 37 °C. For inhibition experiments, 10 μM carbonyl cyanide m-chlorophenylhydrazone (CCCP, Sigma) was included in the washing process. After centrifugation at 800 × g for 2 min, the UVJ mucosa was mounted on a glass slide and was observed under fluorescent microscopy (BX51, Olympus). The images were recorded on a digital camera (DP70; Olympus). The quantitative data were displayed using the fluorescent intensity (FI) ratio (590 nm/530 nm). The fluorescence intensity of the SST or surface epithelium was digitized as described above.

### ATP assay

For measurements of intracellular ATP, the ejaculated sperm (2 × 10^7^ cells/ml) were incubated with or without L-lactic acid for 10 min. After washing with a sperm extender, the sperm pellet was dissolved in ATP assay reagent (“Cellno” ATP Assay reagent, TOYO B-Net Co) and the fluorescent signal was measured using an ImageQuant^TM^ LAS 500 (GE Healthcare).

### Measurement of ATPase activity

Sperm ATPase activity was determined as described previously^40^. Briefly, ejaculated sperm were washed with an assay buffer containing 120 mM KCl, 10 mM β-glycerophosphate, 1 mM DTT, 1.8 mM MgSO_4_ and 10 μM CCCP buffered at pH 7.0 with 10 mM HEPES, and the plasma membrane of spermatozoa was removed by incubating with an assay buffer containing 0.1% Triton X-100 for 2 min. After washing with the assay buffer, the sperm were suspended in the assay buffer with or without L-lactic acid. In the positive control, 10 μM oligomycin or 20 μM sodium vanadate was included in the assay buffer. ATP (final concentration: 1 mM) was added to the incubation mixture and was incubated for 30 min at 39 °C. The reaction was terminated by the addition of ice-cold TCA. The free phosphoric acid in the incubation mixture was colorized by the addition of ammonium molybdate and ferrous sulfate in the presence of sulfuric acid, and the optical density of the solution, at 650 nm measured by a micro plate reader (Tecan Japan).

### Axoneme sliding assay

The demembraned sperm were suspended in an assay buffer and mounted on a chamber slide of 22 × 22 mm and a 24 × 50 mm coverslip. 10 μg/ml trypsin solution was perfused and stood for 2 min for digestion. After washing with assay buffers containing various concentrations of L-lactic acid, axoneme sliding was induced by perfusion of the assay buffer containing 1 mM ATP. Axoneme sliding was observed and recorded with a dark-field microscope (BX51; Olympus) equipped with an immersion dark field condenser (U-DCW) and a 100-W mercury lamp (U-RFL-T). Images were recorded with a high-sensitivity camera (WAT-120N + ; Watec) and captured by iMovieHD (Apple Inc.) with a video frame, and the disintegration of the axoneme was visually analyzed. The number of disintegrated axoneme was counted and the percentage of axoneme sliding calculated.

### RT-PCR

Total RNA was extracted from the isolated SST by collagenase digestion with a commercial kit, RNAiso (Takara Biomedicals). DNA was digested by DNase I (Takara Biomedicals), and was reverse transcribed using a ReverTra Ase kit (Toyobo). PCR amplification was performed using specific primer sets for the MCT1 (sense: 5′- GAACCCTGCCTTAACCATGA - 3′; antisense, 5′- GGCACACCCCATTGTAAATC- 3′), MCT2 (sense: 5′- TGTGGCTGGGTCACTTATGA - 3′; antisense, 5′- CTGGCCACAATCACAATCAC- 3′), MCT3 (sense: 5′- CTGGGTATGGCGTTGAACTT - 3′; antisense, 5′- TAAGTAGGACCAGCCCGATG- 3′) or MCT4 (sense: 5′- AGCACCAAGCCAATAGCACT - 3′; antisense, 5′- GGGTCTGGCACTCAACTTTC- 3′). In parallel, primers (sense: 5′- GACGAAGACGGTGAAGAAGG-3′; antisense, 5′-CTTGGTGTCTGGGTCCACTT-3′) for quail S17 ribosomal protein were used as an internal control. For non-RT control, total RNA was treated in the same way except to replace the reverse transcriptase with water. The products were analyzed on a 1.0% agarose gel stained with ethidium bromide and visualized under UV transillumination and the intensity of the band was quantified by ImageJ software.

### *In situ* hybridization

The frozen sections of the isolated UVJ were prepared for *in situ* hybridization, as described elsewhere^41^. The antisense oligonucleotide probes for *MCT4* (5′- CGCAAAGGAACACTGGGCTCCCAGCAGCAGACAACCCGTTGGCCA -3′, 5′- ATTGCTTCTGCCAGGAGCACCAAGCCGATAGCACTG -3′, 5′- AAACGCCGTGCGGGTTTTGTGCCGTGCTCACACACA -3′, 5′- TGTGACCAGCTGAGGGCAGTGCCTGCGATGTGCCTTTC -3′) were labeled with [^33^P] dATP (NEN Life Science Products) using terminal deoxyribonucleotidyl transferase (Gibco). Hybridization was performed overnight at 42 °C. Washing was performed twice at room temperature for 30 min and at 55 °C for 40 min. After washing, the slides were exposed to X-ray film (Fujifilm) and observed under a stereomicroscope (M165FC; Leica microsystems).

### Library preparation, RNA sequencing and *de novo* assembly and differential expression analysis

Strand-specific libraries for RNA sequencing (RNA-seq) were generated using the SureSelect strand-specific RNA library prep kit (Agilent Technologies). Library preparation was performed according to the manufacturer’s instructions. Total RNA (400 ng) was poly(A)-enriched and then chemically-fragmented to a size appropriate for RNA-Seq library preparation. Fragmented RNA was reverse-transcribed into double-stranded cDNA in the presence of Actinomycin D (Sigma) during first-strand synthesis. Double-stranded cDNA was end-repaired and adenylated before ligation to the SureSelect Oligo Adaptor. The adaptor-ligated cDNA was selectively amplified in PCR with three primers that included the appropriate indexing primer to create the final cDNA library. Libraries of the non-SST cells and SSTs were sequenced (76-bp, paired-end) on the Illumina MiSeq platform.

The raw reads were cleaned up with cutadapt program^42^ by trimming adapter sequences, low-quality ends (QV <30) and the last 76th base, and by discarding reads shorter than 50 bp. Recently, the draft genome of the Japanese quail was sequenced and assembled using next-generation sequencing technology (GenBank Accession number: DRA000595)^43^. We first performed a reference-guided assembly and differential expression analysis using the Tophat and the Cufflinks forour transcriptome data^44^. However, this draft sequence, which was used as the reference genome, did not allow sufficient mapping of our sequence reads to detect differential expression. In addition, comparison of NGS technologies for transcriptome assembly revealed that illumina reads assembled with Trinity produced a high quality transcriptome appropriate for RNA-seq gene expression analyses in the Japanese quail^45^. Therefore, we performed *de novo* assembly of the transcriptome using the Trinity program and used it as a reference for the differential expression analysis. Briefly, the cleaned reads were *de novo* assembled using the Trinity program^46^ version: trinityrnaseq_r20140717. Contaminant sequences derived from rRNA were excluded from the trinity-assembled transcripts by removing sequences matched to the SILVA rRNA database^47^ by megablast. Using scripts included the Trinity plug-in, the cleaned reads were mapped to the rRNA-removed transcripts using Bowtie^48^. Then transcript abundance was estimated using RSEM^49^. Differentially expressed genes between non-SST cells and SSTs were identified using the edgeR package^50^.

We also performed parametric analysis of gene set enrichment (PAGE)^51^ to test the differential expression of gene sets related to glycolysis and hypoxia based on the logFC between the non-SST cells and the SSTs. The GO annotation of the transcriptome was performed using the InterproScan (http://www.geneontology.org/external2go/interpro2go).

### Immunohistochemical detection of hypoxic cells

The laying quails were injected intravenously with a 180 mg/kg body weight of Hypoxyprobe^TM^ (pimonidazole, Natural Pharmacia International) in saline. For control experiments, the same volume of saline was injected. Two hours after injection, the animals were decapitated, the UVJ was removed, and 10 μm frozen sections were prepared for immunohistochemical analysis. The slides were fixed with ice-cold acetone, and immunohistochemistry was performed as suggested by the manufacturer. The samples were stained with 1 μg/ml 4′,6-diamidino-2-phenylindole (DAPI) for 10 min. The immunolabeled sections were examined under a fluorescence microscope (BX51; Olympus).

### *In vitro* storage of the ejaculated sperm

The ejaculated sperm were suspended in sperm extender supplemented with or without 10 mM L-lactic acid. To remove the dissolved oxygen from the medium, the medium was bubbled with nitrogen gas for 5 min before the addition of the sperm. The dissolved oxygen concentration in each medium was measured with a dissolved oxygen meter (InLab OptiOx, Mettler toledo) and the medium with or without the bubbling was 0.95 ± 0.29 or 8.31 ± 0.02 ppm, respectively (n = 5, means ± SD). The sperm suspension was stored for 5 days at 41.5 °C. After the storage, the sperm were fixed with 3.7% formalin and smeared on a glass slide. The sperm morphology was observed under microscope (BX51; Olympus).

### Data analysis

Data were analyzed for significant differences by Student’s t-test. For percentage data, an arcsine square-root transformation was performed and the transformed data was compared by Student’s t-test. The motility score comparisons between groups were made using the Mann-Whitney U test. Differences were considered statistically significant when P < 0.05.

## Additional Information

**How to cite this article**: Matsuzaki, M. *et al.* Lactic acid is a sperm motility inactivation factor in the sperm storage tubules. *Sci. Rep.* **5**, 17643; doi: 10.1038/srep17643 (2015).

## Supplementary Material

Supplementary Information

Supplementary movie1

Supplementary movie2

Supplementary movie3

Supplementary movie4

Supplementary movie5
