# Supplementary material for: Lactic acid is a sperm motility inactivation factor in the sperm storage tubules
Source: Sci Rep. 2015 Dec 1;5:17643. doi: 10.1038/srep17643 (PMC4664960; doi:10.1038/srep17643)
Supplement: Supplementary Information [file srep17643-s1.pdf]

Lactic acid is a sperm motility inactivation factor in the sperm storage tubules

Mei Matsuzaki[1, 2], Shusei Mizushima[1], Gen Hiyama[1], Noritaka Hirohashi[3], Kogiku Shiba[4], Kazuo Inaba[4], Tomohiro Suzuki[5], Hideo Dohra[5], Toshiyuki Ohnishi[1], Yoshikatsu Sato[7], Tetsuya Kohsaka[1] Yoshinobu Ichikawa[1] Yusuke Atsumi[6], Takashi Yoshimura[6, 7] and Tomohiro Sasanami[1 *]

[1]Department of Applied Biological Chemistry, Faculty of Agriculture, Shizuoka University, 836 Ohya, Shizuoka, Shizuoka 422-8529, Japan; [2]United Graduate School of Agricultural Science, Gifu University, 1-1 Yanagido, Gifu 501-1193, Japan; [3]Oki Marine Biological Station, Education and Research Center for Biological Resources, Faculty of Life and Environmental Science, Shimane University, 194 Kamo, Okinoshima-cho, Oki, Shimane 685-0024, Japan; [4]Shimoda Marine Research Center, University of Tsukuba, 5-10-1 Shimoda, Shizuoka 415-0025, Japan; [5]Research Institute of Green Science and Technology, Shizuoka University, 836 Ohya, Shizuoka, Shizuoka 422-8529, Japan; [6]Graduate School of Bioagricultural Sciences, Nagoya University, Furo-cho, Chikusa-ku, Nagoya 464-8601, Japan; [7]Institute of Transformative Bio-Molecules (WPI-ITbM), Nagoya University, Furo-cho, Chikusa-ku, Nagoya 464-8601, Japan

*Correspondence to: Tomohiro Sasanami, Department of Applied Biological Chemistry, Faculty of Agriculture, Shizuoka University, 836 Ohya, Shizuoka, Shizuoka 422-8529, Japan. Email: atsasan@ipc.shizuoka.ac.jp

Supplementary Information

Supplementary Table S1

Supplementary Figure S1: Expression of monocarboxylate transporter 4 (MCT4) in the utero-vaginal junction (UVJ).

Supplementary Figure S2: Effects of various acids treatment on sperm motility.

Supplementary Figure S3: Effects of oligomycin and sodium vanadate on ATPase activity of sperm.

Supplementary Figure S4: Effects of in vitro storage on the morphology of spermatozoa.

Caption to Supplementary Movie 1: Effects of a flow-through fraction of utero vaginal junction (UVJ) extracts on sperm motility.

Caption to Supplementary Movie 2: Sperm motility in Hank's balanced salt solution (HBSS).

Caption to Supplementary Movie 3: Effects of L-lactic acid and D-lactic acid on sperm motility.

Caption to Supplementary Movie 4: Axonemal sliding of de-membraned sperm in a buffer at pH 7.4.

Caption to Supplementary Movie 5: Axonemal sliding of de-membraned sperm in a buffer at pH 5.4.

Supplementary Table S1

Enrichment of gene ontology terms in differentially expressed sequences between SSTs and non-SST cells detected by PAGE

GO_id	GO_name	Number of sequences	Z score	P-value	False discovery rate
GO:0005515	MF protein binding	2354	-7.794049752	6.43929E-15	1.71E-12
GO:0005840	CC ribosome	98	6.548828012	5.79903E-11	5.93E-09
GO:0003735	MF structural constituent of ribosome	99	6.527433457	6.69063E-11	5.93E-09
GO:0006412	BP translation	99	6.373236731	1.8508E-10	1.23E-08
GO:0005576	CC extracellular region	110	6.049948334	1.44892E-09	7.71E-08
GO:0006820	BP anion transport	20	-5.426567452	5.74481E-08	2.55E-06
GO:0005524	MF ATP binding	929	-5.361703161	8.24409E-08	3.13E-06
GO:0008289	MF lipid binding	21	5.334001178	9.60719E-08	3.19E-06
GO:0003777	MF microtubule motor activity	38	-4.870426596	1.11358E-06	3.29E-05
GO:0007018	BP microtubule-based movement	39	-4.827499659	1.38258E-06	3.44E-05
GO:0003774	MF motor activity	58	-4.804414293	1.55205E-06	3.44E-05
GO:0016459	CC myosin complex	58	-4.804414293	1.55205E-06	3.44E-05
GO:0006468	BP protein phosphorylation	423	-4.718625472	2.37444E-06	4.86E-05
GO:0017048	MF Rho GTPase binding	12	-4.468511328	7.87658E-06	0.000149655
GO:0005622	CC intracellular	367	4.357143873	1.31771E-05	0.000233674
GO:0004672	MF protein kinase activity	415	-4.332896983	1.4716E-05	0.000244654
GO:0005262	MF calcium channel activity	10	-4.269214999	1.96162E-05	0.000306936

GO:0007156	BP homophilic cell adhesion via plasma membrane adhesion	53	-4.232014107	2.31608E-05	0.000342265
GO:0005882	CC intermediate filament	31	4.116758099	3.84239E-05	0.000537935
GO:0070588	BP calcium ion transmembrane transport	10	-4.036394977	5.42788E-05	0.000721908
GO:0005856	CC cytoskeleton	42	-3.603256105	0.000314256	0.003980576
GO:0016311	BP dephosphorylation	20	-3.210644436	0.001324377	0.016012922
GO:0004198	MF calcium-dependent cysteine-type endopeptidase activity	13	-3.162832965	0.00156242	0.017429501
GO:0030036	BP actin cytoskeleton organization	18	-3.152004905	0.001621535	0.017429501
GO:0005737	CC cytoplasm	221	3.149034063	0.001638111	0.017429501
GO:0008017	MF microtubule binding	36	-3.130481343	0.001745201	0.017854749
GO:0015074	BP DNA integration	13	-3.091820668	0.00198933	0.019598584
GO:0004867	MF serine-type endopeptidase inhibitor activity	18	3.072754654	0.002120928	0.019921593
GO:0004871	MF signal transducer activity	46	-3.065659791	0.002171903	0.019921593
GO:0005179	MF hormone activity	13	3.000344938	0.00269674	0.023911095
GO:0008236	MF serine-type peptidase activity	21	2.976241845	0.002918046	0.025038717
GO:0008146	MF sulfotransferase activity	34	2.892907378	0.003816938	0.031728297
GO:0005509	MF calcium ion binding	269	-2.864577588	0.004175658	0.032881818
GO:0019001	MF guanyl nucleotide binding	15	-2.853316217	0.004326555	0.032881818
GO:0031683	MF G-protein beta/gamma-subunit complex binding	15	-2.853316217	0.004326555	0.032881818
GO:0016791	MF phosphatase activity	25	-2.831161577	0.004637929	0.034269142
GO:0005198	MF structural molecule activity	60	2.801202112	0.005091262	0.035857906

GO:0006810	BP transport	127	-2.799224055	0.005122558	0.035857906
GO:0008237	MF metallopeptidase activity	18	-2.79045819	0.00526335	0.035898746
GO:0008168	MF methyltransferase activity	63	2.775133315	0.005517908	0.036694088
GO:0030286	CC dynein complex	12	-2.752165169	0.005920265	0.038409524
GO:0005215	MF transporter activity	54	-2.736886144	0.006202376	0.039281715
GO:0008233	MF peptidase activity	17	2.727139113	0.00638861	0.039520239
GO:0016491	MF oxidoreductase activity	200	2.706477648	0.006800116	0.041109792
GO:0016020	CC membrane	689	-2.668275556	0.00762417	0.045067316
GO:0036459	MF ubiquitinyl hydrolase activity	51	-2.616318016	0.008888373	0.051397983
GO:0006457	BP protein folding	51	2.565771027	0.010294676	0.058263485
GO:0004129	MF cytochrome-c oxidase activity	15	2.515646606	0.011881425	0.065842897
GO:0004553	MF hydrolase activity, hydrolyzing O-glycosyl compounds	39	-2.383112906	0.017166928	0.093191895
GO:0006629	BP lipid metabolic process	37	2.279286886	0.022650017	0.12049809
GO:0045454	BP cell redox homeostasis	43	2.243244467	0.024881054	0.129771772
GO:0004866	MF endopeptidase inhibitor activity	10	-2.23035619	0.025723806	0.131587161
GO:0006814	BP sodium ion transport	25	2.215630772	0.0267168	0.134088091
GO:0006281	BP DNA repair	65	2.142772777	0.032131348	0.15827664
GO:0005085	MF guanyl-nucleotide exchange factor activity	25	-2.106623274	0.035150248	0.167675308
GO:0005507	MF copper ion binding	18	2.097576928	0.035942535	0.167675308
GO:0030001	BP metal ion transport	19	-2.079932185	0.037531753	0.167675308

GO:0003723	MF RNA binding	128	2.079504008	0.03757105	0.167675308
GO:0005525	MF GTP binding	280	2.078047394	0.037704994	0.167675308
GO:0016887	MF ATPase activity	87	-2.076784014	0.037821498	0.167675308
GO:0042626	MF ATPase activity, coupled to transmembrane movement of substances	28	-2.039379711	0.041412146	0.176095407
GO:0008083	MF growth factor activity	31	2.037561024	0.041593853	0.176095407
GO:0003779	MF actin binding	63	-2.036433876	0.041706807	0.176095407
GO:0006418	BP tRNA aminoacylation for protein translation	37	1.999249884	0.045581324	0.189447378
GO:0006470	BP protein dephosphorylation	85	-1.988998362	0.046701384	0.190822657
GO:0009058	BP biosynthetic process	52	1.98318315	0.047346975	0.190822657
GO:0006955	BP immune response	31	1.967460774	0.049130114	0.193423393

Notes: GO, gene ontology; PAGE, parametric analysis of gene set enrichment; BP, biological process; MF, molecular function; and CC, cellular component. We used log2 Fold Change values between SSTs and non-SST cells to calculate Z scores and corresponding P-values for each GO term.

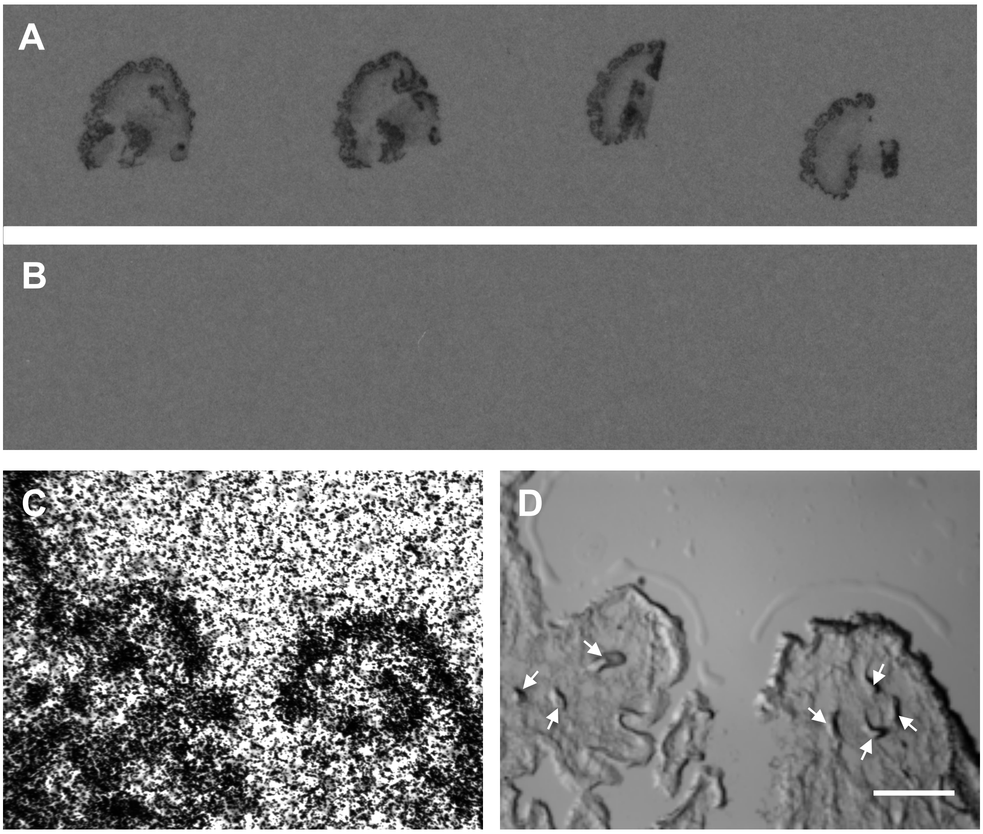

Supplementary Figure S1: Expression of *monocarboxylate transporter 4* (*MCT4*) mRNA in the utero-vaginal junction (UVJ). a-b, Autoradiograms of the UVJ sections after hybridization with ^{33}P-labeled antisense probe specific for *MCT4* (**a**) or sense probe (**b**) are shown. The autoradiogram was observed under a stereomicroscope (**c**) and the corresponding area of the glass slide was photographed (**d**). Arrows in panel **d** indicate SSTs. Note that the corresponding areas of the autoradiogram in panel **c** are positively labeled. Representative results of two experiments are shown (n= 2). Scale bar = 200 μm.

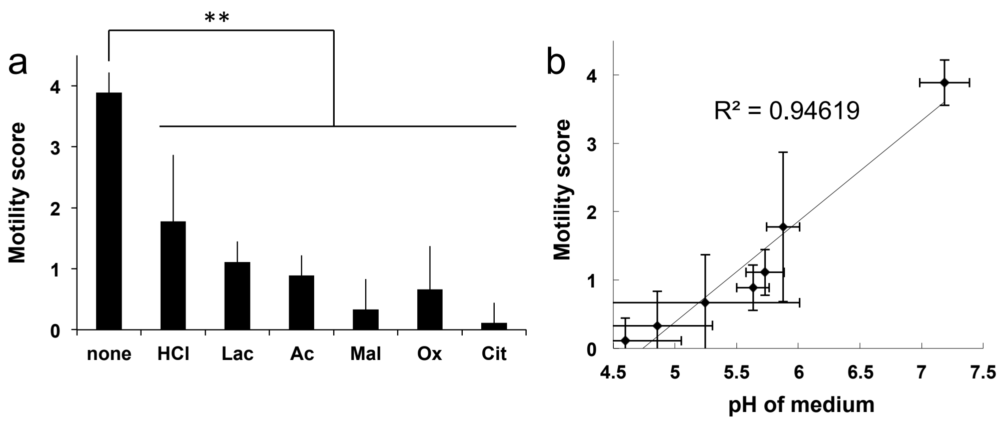

Supplementary Figure S2: Effects of treatment with various acids on sperm motility. Ejaculated sperm were incubated with various organic acids including lactic acid (Lac), acetic acid (Ac), malic acid (Mal), oxaloacetic acid (Ox) and citric acid (Cit) at a final concentration of 10 mM. In controls, the effects of medium alone (none) and non-organic acid (HCl) were also examined. **a,** Sperm motility was scored (n=3) after 10 min incubation of sperm with these media. ** denotes a significant difference from medium alone ($P < 0.01$). **b,** The relationship between medium pH (x-axis) and motility score (y-axis) was indicated (n=3).

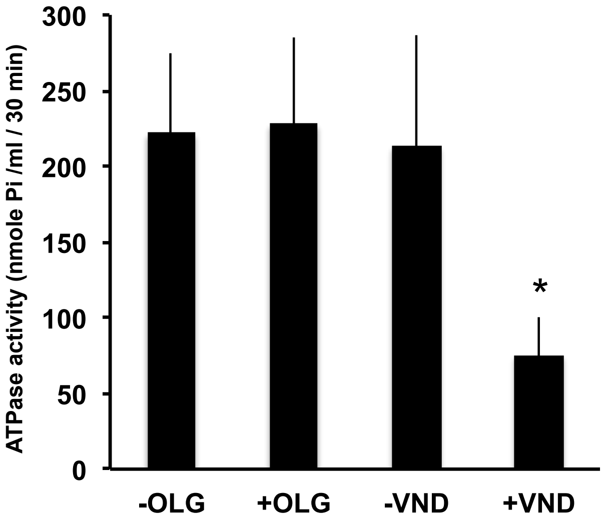

Supplementary Figure S3: Effects of oligomycin and sodium vanadate on ATPase activity of sperm. De-membraned sperm were incubated with 1 mM ATP in the presence or absence of 10 µM oligomicin (OLG) or 20 µM sodium vanadate (VND), and the free phosphoric acid in the incubation mixture was measured using a microplate reader. Values are mean ± SEM of three independent experiments. * denotes a significant difference from respective control ($P < 0.05$).

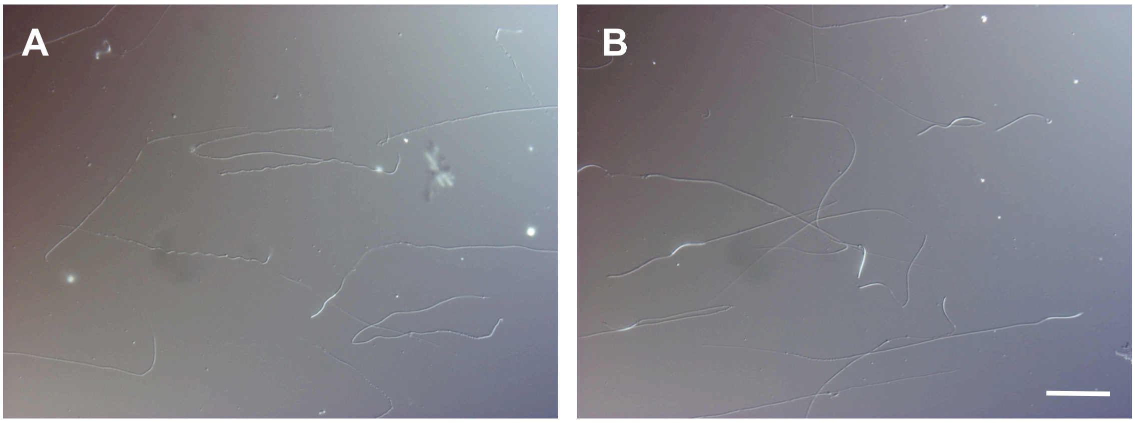

Supplementary Figure S4: Effects of in vitro storage on the morphology of spermatozoa. Ejaculated sperm were suspended in either a medium alone (*a*) or a medium containing 10 mM L-lactic acid with nitrogen gas bubbled through it for 5 min (*b*). After being stored for 5 days at 41.5°C, sperm were fixed with 3.7% formalin and smeared on a glass slide. Sperm morphology was observed under a microscope. The images shown are representative of images obtained from three independent experiments. Scale bar = 50 μm.

Supplementary Movie 1. Effects of a flow-through fraction of utero vaginal junction (UVJ) extracts on sperm motility. UVJ extracts were passed through an ultrafiltration membrane (MW cutoff 10 kDa) and the flow-through fraction was examined using a sperm motility assay. Sperm movement was recorded using a high-speed camera (200 frames per second), which showed that sperm motility was strongly suppressed by the addition of the fraction.

Supplementary Movie 2. Sperm motility in Hank's balanced salt solution (HBSS). Ejaculated sperm were suspended in HBSS and sperm movement was recorded using a high-speed camera (200 frames per second).

Supplementary Movie 3. Effects of L-lactic acid and D-lactic acid on sperm motility. Ejaculated sperm were suspended in Hank's balanced salt solution and 10 mM L-lactic acid or D-lactic acid was injected into sperm suspensions using a glass capillary. Sperm movement was recorded under a stereomicroscope.

Supplementary Movie 4. Axonemal sliding of de-membraned sperm in a buffer at pH 7.4. Vigorous axonemal sliding was observed subsequent to perfusion of the ATP solution.

Supplementary Movie 5. Axonemal sliding of de-membraned sperm in a buffer at pH 5.4. Little axonemal sliding was observed subsequent to perfusion of the ATP solution.
